# Development of a Non-Meat-Based, Mass Producible and Effective Bait for Oral Vaccination of Dogs against Rabies in Goa State, India

**DOI:** 10.3390/tropicalmed4030118

**Published:** 2019-09-04

**Authors:** Andrew D. Gibson, Stella Mazeri, Gowri Yale, Santosh Desai, Vilas Naik, Julie Corfmat, Steffen Ortmann, Alasdair King, Thomas Müller, Ian Handel, Berend MdeC. Bronsvoort, Luke Gamble, Richard J. Mellanby, Ad Vos

**Affiliations:** 1 Mission Rabies, Cranborne, Dorset BH21 5PZ, UK (S.M.) (L.G.); 2 The Royal (Dick) School of Veterinary Studies and the Roslin Institute, Easter Bush Campus, The University of Edinburgh, Roslin, Midlothian EH25 9RG, UK (I.H.) (B.M.B.) (R.J.M.); 3 Mission Rabies, Tonca, Miramar, Panjim, Goa 403002, India (G.Y.) (J.C.); 4 Department of Animal Husbandry and Veterinary Services, Government of Goa, Panjim, Goa 403001, India (S.D.) (V.N.); 5 IDT Biologika GmbH, 06861 Dessau, Rosslau, Germany (S.O.) (A.V.); 6 Merck Animal Health, Madison, NJ 07940, USA; 7 Friedrich-Loeffler-Institut, Federal Research Institute for Animal Health, WHO Collaborating Centre for Rabies Surveillance and Research, 17493, Greifswald, Insel Riems, Germany

**Keywords:** rabies, free-roaming dogs, oral vaccination, bait, sachet

## Abstract

*Introduction:* To achieve the global goal of canine-mediated human rabies elimination by 2030 there is an urgent need to scale-up mass dog vaccination activities in regions with large dog populations that are difficult to access; a common situation in much of India. Oral rabies vaccination may enable the vaccination of free-roaming dogs that are inaccessible to parenteral vaccination, and is considered a promising complementary measure to parenteral mass dog vaccination campaigns. WHO and OIE have published detailed minimum requirements for rabies vaccines and baits to be used for this purpose, requiring that baits must not only be well-accepted by the target population but must also efficiently release the vaccine in the oral cavity. For oral rabies vaccination approaches to be successful, it is necessary to develop baits which have a high uptake by the target population, are culturally accepted and amenable to mass production. The aim of this study was to compare the interest and uptake rates of meat-based and an egg-based prototype bait constructs by free roaming dogs in Goa, India. *Methods:* Three teams randomly distributed two prototype baits; an egg-flavoured bait and a commercial meat dog food (gravy) flavoured bait. The outcomes of consumption were recorded and compared between baits and dog variables. *Results*: A total of 209 egg-bait and 195 gravy-bait distributions were recorded and analysed. No difference (*p* = 0.99) was found in the percentage of dogs interested in the baits when offered. However, significantly more dogs consumed the egg-bait than the gravy-bait; 77.5% versus 68.7% (*p* = 0.04). The release of the blue-dyed water inside the sachet in the oral cavity of the animals was significant higher in the dogs consuming an egg-bait compared to the gravy-bait (73.4% versus 56.7%, *p* = 0.001). *Conclusions*: The egg-based bait had a high uptake amongst free roaming dogs and also enabled efficient release of the vaccine in the oral cavity, whilst also avoiding culturally relevant materials of bovine or porcine meat products.

## 1. Introduction

An ambitious goal of eliminating dog-mediated human rabies by 2030 has been set by the tripartite; World Health Organisation (WHO), World Organisation for Animal Health (OIE) and the Food and Agriculture Organization of the United Nations (FAO) [1,2]. Prevention of human deaths from rabies could be achieved through increasing access to post-exposure prophylaxis (PEP), ensuring that anyone bitten by a rabid dog received comprehensive post-exposure prophylaxis. However, this approach fails to resolve the problem at its source since almost all human rabies deaths follow a bite from a rabies virus-infected dog. Focusing only on the human component of rabies prevention will result in the need for indefinite distribution of human PEP whilst the risk of infection remains from the animal population [3]. Mass dog vaccination reaching over 70% of the dog population has been demonstrated to be the most cost-effective approach to rabies prevention through elimination of the virus in the reservoir population [4].

In several settings in Africa, it has been shown that the majority of free roaming dogs are owned and thus, high vaccination coverage can be achieved through mobilizing dog owners to present their dogs at static vaccination locations or to roaming vaccination teams travelling door-to-door, described as central point (CP) or door-to-door (DD) methods respectively [5,6,7]. However, in countries such as India, a large proportion of free-roaming dogs are either owned, but cannot be readily handled by their owner, or are truly ownerless and cannot be easily restrained by anyone in the local community [8,9]. These dogs, which cannot be handled by vaccinators for parenteral vaccination without special equipment, are considered “inaccessible”. In such settings, DD and CP approaches do not access a high enough proportion of dogs to interrupt rabies virus transmission and therefore, there is a clear need to develop novel approaches to vaccinate inaccessible dogs.

It is possible to increase vaccination coverage in inaccessible dog populations through enhanced methods such as deploying catching teams with nets to capture inaccessible dogs for vaccination. This catch-vaccinate-release (CVR) approach can achieve vaccination coverages in the region of 70% in urban settings, however they require large numbers of staff and high equipment costs in comparison to DD and CP approaches [10,11].

An alternative method for vaccinating inaccessible dogs is through oral rabies vaccination (ORV); offering bait containing a vaccine-loaded sachet. Currently available ORVs consist of modified live rabies virus or recombinant constructs, as opposed to modern parenteral vaccines which consist of inactivated rabies virus [3]. Upon acceptance of the bait, the sachet is perforated by the teeth and the vaccine is released in the oral cavity. Subsequently, the vaccine enters the body, predominantly at the palatine tonsils, and after limited locally restricted replication, induces a protective immune response [12]. This approach has been successfully developed for several rabies reservoir species including red fox (*Vulpes vulpes*), raccoon dog (*Nyctereutes viverrinus*), coyote (*Canis latrans*), gray fox (*Urocyon cinereoargenteus*), raccoon (*Procyon lotor*) and golden jackal (*Canis aureus*) [13,14,15,16]. For example, in Germany, the number of rabies cases in animals fell from 10,487 in 1983 to 83 cases in 1997 and ultimately leading to ‘rabies-free’ status in 2008 following sustain systematic vaccination of the reservoir fox population using ORV [17], with the European Union aiming to eliminate fox-mediated rabies by 2020 [18].

Studies in recent decades have indicated that ORV of dogs may be of considerable benefit to increasing vaccination coverage within inaccessible dog populations [19], however there is yet to be a large scale example of its implementation. OIE and WHO have advocated for its incorporation into mass dog vaccination initiatives, following evaluation in settings where they could be of benefit to rabies control [3,20]. A recent study in Goa state investigated the oral bait handout method (OBH), whereby baits are offered to inaccessible dogs individually and unconsumed baits and discarded perforated sachets are re-collected. The OBH method was combined with the DD parenteral vaccination of accessible dogs. When compared with the existing CVR vaccination protocol, the OBH method was more efficient both in terms of human resource and estimated cost per dog vaccinated [21].

Identifying a bait that is well accepted by the local dog population is crucial to the success of ORV of dogs. The smell, palatability, texture, shape and size of the bait construct is critical to ensuring uptake of the vaccine sachet into the dog’s mouth and chewing to ensure perforation and release of the liquid contents into the oral cavity before the remnants are swallowed or discarded. Many studies have demonstrated wide variation in bait preferences between dog populations of different countries [22,23,24,25,26,27,28], however, comparison of results between studies is challenging due to variation in study design and bait type.

Previous studies in the Philippines, Turkey, the Navajo Nation, Haiti and Thailand showed that baits made by placing the vaccine sachet inside locally available bovine or porcine intestine were not only very attractive for dogs but were also efficient in delivery the vaccine in the oral cavity [27,28,29,30,31,32]. This intestine bait is not suitable for use at scale in India due to difficulty in mass production and as well as the need to avoid bovine and porcine meat products for its use to be broadly acceptable in Hindu and Islamic communities [33]. Therefore, alternative solutions for mass producible bait constructs have been explored, which have included fish, commercial pet food and egg-based baits [29,30]. Pet food-based and egg-based baits were considered most feasible for mass production and so were selected for use in this study.

This study aimed to compare the acceptance, perforation and swallowing rates of two bait constructs by roaming local breed dogs in Goa, India. The study design is comparable to that used in previous studies conducted in the Navajo Nation and in Thailand [29,30]. The results showed that ORV of dogs is also a feasible option for India as similar high bait acceptance rates were observed.

## 2. Materials and Methods

### 2.1. Study Area

Mission Rabies is a non-governmental organisation (NGO) working with international partners, local governments and NGOs to develop effective methods for mass dog vaccination campaign implementation and rabies control at the ground level [34]. The study was conducted in Goa State, India as part of the ongoing rabies control activities being conducted by Mission Rabies in partnership with the Government of Goa to investigate alternative methods for accessing dogs for rabies vaccination. Locations were chosen at random within two urban regions: Panjim and Goa Velha.

### 2.2. Bait Constructs

**Figure 1:**
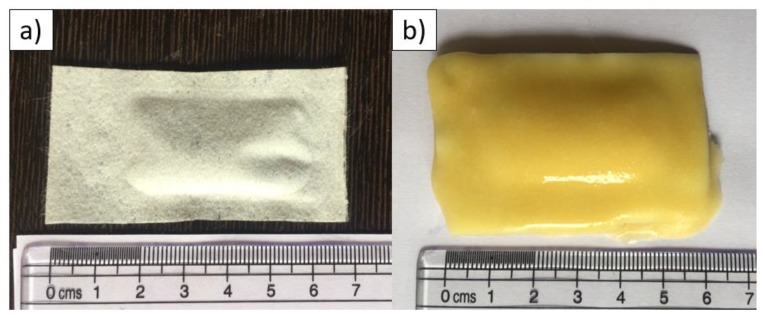
Photographs of the two bait construct types; (**a**) gravy-bait (before being dipped in dog food gravy) and (**b**) egg bait.

The highly attractive baits made from porcine or bovine intestine used in previous studies would likely not be culturally accepted across India and hence were not evaluated. Two bait constructs were tested; the placebo vaccine sachet was incorporated in an egg-flavoured bait matrix (‘egg-bait) or coated with a commercially available pet food gravy (‘gravy-bait’) (Figure 1).

#### 2.2.1. Capsule

During previous screening trials in the study area it was observed that the sachets, PVC-capsules sealed (‘hard blister’) with aluminum foil, used in the Navajo Nation and Thailand were often swallowed [35]. It was decided to replace these sachets for this trial with a sachet made from biodegradable foil covered with a fleece that can absorb fluids; ‘soft blister’ (70 × 35 × 5 mm). The sachets contained a food colorant (Patent Blue V, Thermo Fischer Scientific, Geel, Belgium) and sucrose dissolved in water (3 mL) and no active ingredients. The colorant was added to increase detectability if the contents of the sachet were released in the oral cavity.

#### 2.2.2. Egg-Bait

The industrial manufactured egg-flavoured bait was almost identical to the baits used in previous studies (proprietary to IDT Biologika, Dessau-Rosslau, Germany) [29,30]. Due to import restrictions, the egg-baits were manufactured in India using locally available materials that partially deviated from the original ingredients and method, meaning that the egg matrix coating differed in thickness and flavour to previous baits. After preparation, batches of 15 egg-baits were placed in a foil zip-bag and stored frozen until used.

#### 2.2.3. Gravy-Bait

The outer layer of the sachet used in this study is absorptive to liquid. Therefore, batches of 15 sachets were placed in a zip-bag and at the beginning of each distribution session shortly before use, 100 g of commercially available pet food gravy (chicken flavour) was poured into the zip-bag, coating the sachets and soaking into the outer layer of the sachet.

## 3. Study Design and Bait Distribution

The field study took place on the 11th and 12th of July 2018. Three teams, each consisting of two people travelled by moped distributing baits in randomly allocated sections of the study area between 07:00 and 17:00. Baits were defrosted shortly before each vaccination session and carried by the teams in separate bags within portable cool boxes. One person was responsible for offering the bait to the dog and the other for data collection. The type of bait offered to each dog was randomly pre-determined and free-roaming stray dogs were the target population for bait administration.

Staff were trained in bait distribution methods as in a previous study of OBH methods in Goa State [21]. Briefly, staff were trained to approach dogs indirectly, avoiding eye-contact, dropping the bait in front of the dog whilst continuing to walk on and having the data collector watch the dog inconspicuously from a distance, recording their observations. The training consisted of a class-room based session, entering dummy data under supervision into the forms based on observing video footage of bait administrations, followed by a supervised field session. The whole training was no more than three hours in duration. When dogs were in a group, baits were distributed individually to spread out the dogs and minimise competition, with the dominant animal being offered first. Any remnants of baits discarded by dogs were recollected by the observer and not left behind. A project contact number was distributed for members of the public to contact for any questions about the study.

## 4. Data Collection

Data about every bait dispensed were recorded in customized forms in the WVS App [36]. This automatically captured the GPS location, time, date and user of each data point. Compulsory fields were then displayed for entering information about the bait interaction and about the dog. These fields were comparable to data recorded during the field studies in Thailand and the Navajo Nation [28,29].

Data recorded about the bait interaction included the type of bait (egg/gravy), acceptance, consumption, sachet perforation, bait handling time, bait outcome and bait efficacy. Bait acceptance was defined as whether the dog showed interest with direct oral/nasal contact with the bait through sniffing or licking (‘interested’), or ignored or did not acknowledge the bait (‘ignored’). Consumption was recorded as whether the dog took the bait into the mouth or not and for egg baits what proportion of the egg casing was consumed (<50%, >50%, 100%). Perforation was recorded on whether the sachet was observed to be perforated by the teeth of the dog. Bait handling time was estimated based on the observed time that the dog manipulated (chewed) the bait (<10 s, 10–30 s, 30–60 s or >60 s) and the outcome of whether the sachet remnants were swallowed or discarded was also recorded. Finally, bait efficacy was recorded as the staff member’s assessment of whether the dog would have been effectively vaccinated if the sachet contained active oral rabies vaccine, based on observing release of the blue dyed-water in the oral cavity. For all answers an “Unknown” field was available if the outcome was not observed, for example if the dog took the bait out of sight.

Data recorded about each dog offered bait included whether the dog was alone (‘single’) or with other dogs (‘multiple’) and if so, how many other dogs were present, age (adult/juvenile/puppy), sex (male/female) and size (small/medium/large).

## 5. Statistical Analysis

The data from the WVS App were downloaded in comma-separated values (CSV) format. Further analysis was then performed in R statistical software environment [37].

Chi squared test was used to identify association between individual variables.

Multivariable logistic regression was used to estimate the difference in bait acceptance between the egg bait and the gravy bait, adjusting for other factors including dog size, presence of multiple dogs, age, time of day, sex, and team. Two different models were built. The outcome variable of the first model was whether, according to the data collector, blue dyed liquid was released in the oral cavity, i.e., likely theoretical “vaccination” if ORV had been present. The outcome variable of the second model was possible “vaccination”, i.e., dogs who were seen to release the blue-dyed liquid in the oral cavity as well as those where the bait was seen to have been taken in the mouth of the dog, but the perforation status of the capsule was not confirmed within the oral cavity were both classed as “vaccinated”. All predictors were considered for inclusion in the model. All possible variable combinations were considered using the package *MuMIn* [38]. The model with lowest Akaike information criterion (AIC) was chosen as the final model.

Ordinal logistic regression was used to evaluate the effect of presence of multiple dogs and team on bait handling time.

Results were plotted using package ggplot2 [39].

## 6. Ethical Statement

Permission for the study was granted by the Department of Animal Husbandry and Veterinary Services, Government of Goa. Ethics approval was provided by University of Edinburgh R(D)SVS Veterinary Ethical Review Committee (Reference number 113.18).

## 7. Results

A total of 406 baits were recorded, however two were removed, one because it was recorded as bait type ‘unknown’ and one bait was taken by birds, leaving a total of 404 samples (209 egg-bait, 195 gravy-bait). Eight records reported bait acceptance as ‘ignored’, but the bait was also recorded as ‘consumed’ with subsequent information about vaccine release and chewing time, therefore, bait acceptance was corrected to ‘interested’. One record was recorded as ‘not consumed’, but also the theoretical vaccination status as ‘vaccinated’ and so this record was amended to ‘not vaccinated’. In six cases (two egg-bait and four gravy-bait), the perforation status was recorded as ‘unknown’, whilst the theoretical vaccination status was ‘vaccinated’, so the vaccination status was amended to ‘unconfirmed’.

**Figure 2:**
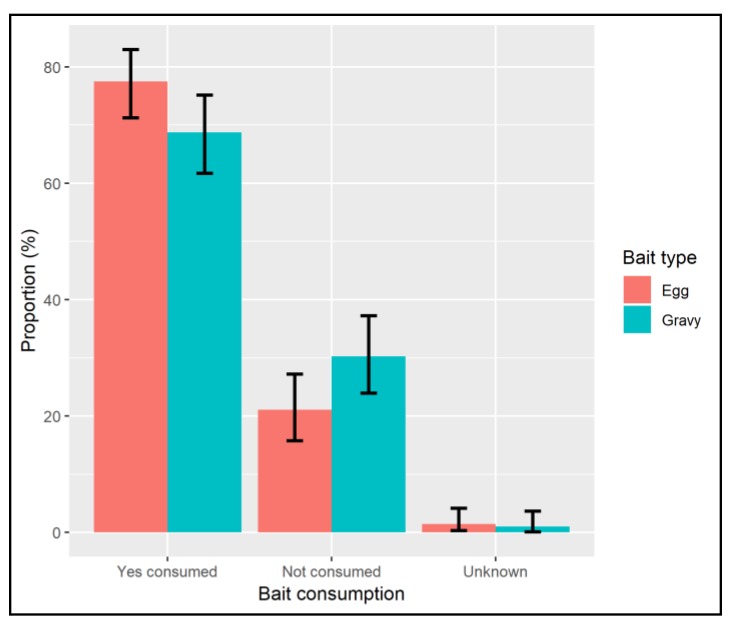
Chart showing the proportion of all dogs offered baits which took the bait into the oral cavity (consumed).

Overall, a high proportion of dogs showed an interest in both bait types, with 81.3% (CI: 75.4–86.4%) and 81.0% (CI 74.8–86.3%) of dogs making oral or nasal contact when offered egg and gravy-baits respectively (Supplementary Materials Section 1). A significantly higher proportion of dogs consumed (took the bait into the oral cavity) the egg bait (77.5%, CI: 71.2–83.0%) than did for those offered gravy-baits (68.7%, CI: 61.7–75.2%) (Supplementary Materials Section 2); Chi^2^ = 3.94, df = 1, *p* = 0.047 (Figure 2).

Of the 296 baits consumed, the perforation status of 269 sachets was observed. Of baits where the outcome was observed, the sachet within the egg-bait (91.1%; CI: 86.4–95.7%) was significantly more often perforated than with the gravy-bait (72.4%; CI: 64.4–80.2%); Chi^2^ = 14.98, df = 1, *p* < 0.001 (Supplementary Materials Section 3).

**Figure 3:**
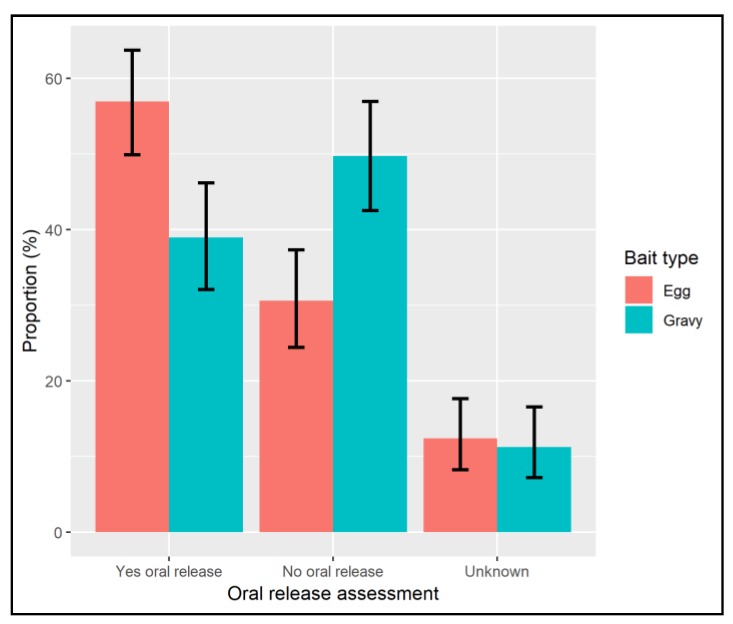
Chart showing the proportion of all dogs offered baits which were observed to release of the blue-dye liquid sachet contents in the oral cavity, therefore likely ‘vaccination’ if vaccine had been present in the liquid.

Theoretical ‘vaccination’ success as indicated by observation of release of the blue-dyed liquid in the oral cavity was more likely in dogs offered an egg-bait than dogs offered a gravy-bait and this effect was significant when adjusting for other factors (OR 2.25, CI 1.47–3.43). Blue-dye liquid was observed as released in the oral cavity in 73.4% of dogs consuming the egg-bait and 56.7% of dogs offered gravy-bait, representing 56.9% and 39.0% of all dogs offered egg and gravy baits respectively (Table 1, Figure 3). The outcome of 30 egg-baits and 24 gravy-baits was recorded as ‘unknown’ and were considered as ‘vaccination’ failures in the estimates above. Oral release may have occurred in these cases and so if they were included, the proportion of all dogs that may have been ‘vaccinated’ was 71.3% of dogs offered egg baits and 51.3% of dogs offered gravy-baits (Table 1).

The multivariable logistic regression model showed no association between dog age, dog size or time of day with bait consumption. Significant differences were observed between the three teams in the proportion of dogs interested, consuming and considered vaccinated after being offered a bait (Supplementary Materials Section 7).

**Figure 4:**
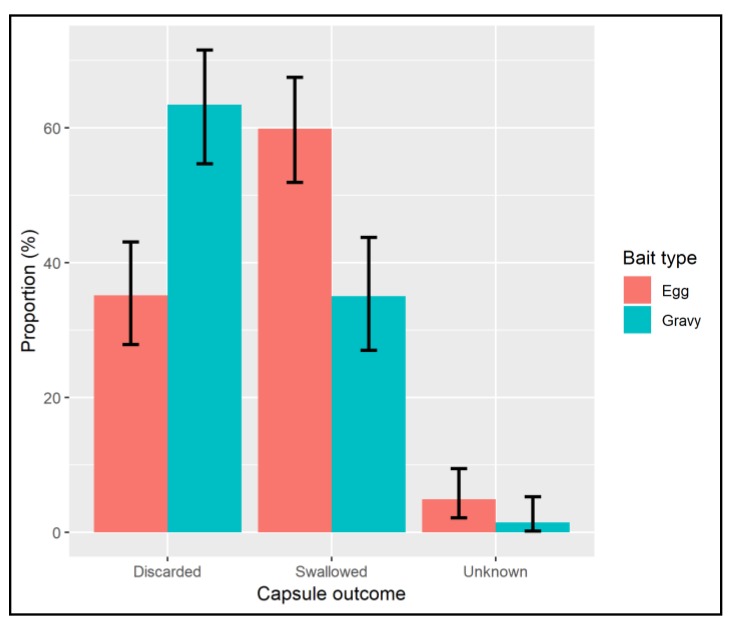
Chart showing the proportion of bait sachets swallowed and discarded for each bait type.

The outcome of the sachet was observed for 286 of the 296 consumed baits. Significantly more sachets were swallowed for the egg-bait (59.9% CI 51.9–67.5%) than were for the gravy-bait (35.1% CI 27.0–43.8%) (Chi^2^ = 20.2, df = 1, *p* < 0.001), with the gravy-bait sachet being more likely to be discarded (Figure 4, Supplementary Materials Section 4).

The duration of bait handling did not differ significantly between the two bait types; Chi^2^ = 4.840, df = 3, *p* = 0.183 (Supplementary Materials Section 8). Single dogs took significantly longer to consume the bait than dogs who were offered a bait when the animals were together with other dogs (Chi^2^ = 12.566, df = 3, *p* = 0.0056). Ordinal logistic regression indicated a difference between chewing time for single dogs versus dogs in a group, however when the team was added as a variable, this effect is not seen, indicating a potential user bias in the recording of bait handling time (Supplementary Materials Section 8). Of baits that were consumed, all of the egg-bait material was eaten in 54.8% (CI: 46.4–63.0%) of cases.

## 8. Discussion

This study investigated the acceptance rates of oral bait constructs in free roaming local breed dogs. The egg-bait was found to be a better candidate than the gravy-bait as a potential vehicle for delivering ORV in the roaming dog population in India.

Just as a safe and efficacious vaccine is important, the identification of a bait that is widely consumed by the target population and releases the vaccine in the oral cavity is prerequisite to the success of ORV. OIE has listed detailed requirements and characteristics of baits to be used for ORV, among others; (1) it should be designed specifically for the target species, (2) it should be attractive to the targeted population (local food preferences), (3) it should remain stable under a wide range of temperatures and weather conditions, (4) it should optimize the release of the vaccine into the targeted tissues (in the oral cavity), (5) its ingredients should be safe for target and non-target species and should comply with animal feed standards and not interfere with vaccine activity, (6) it should allow the incorporation of a biomarker, and (7) feature a labelling system, including public warning and identification of the product [40].

Previous studies have used PVC-based vaccine sachets [24,27,28,29,30], however, there would be concern using such sachets at scale in conjunction with bait constructs that result in high rates of swallowing due to the risk of gastrointestinal tract irritation or obstruction. Therefore, a soft sachet was developed which reduces any risk of gastrointestinal tract obstruction if swallowed. The absorbent outer layer of the soft sachet made it possible to effectively coat the sachet in commercially available pet food gravy, to make the sachet palatable in the case of the gravy-bait and to attach the egg casing for the egg-bait.

Although the egg-bait ingredients, sachet type and bait sizes varied between studies, there were considerable differences in the swallowing rate of the sachets in the egg-bait between India (63%), Thailand (2%) and the Navajo Nation (59%) studies. The Navajo Nation study compared ‘hard’ and ‘soft’ sachet types in numerous bait constructs, with soft sachet type baits having significantly higher swallow rates than those containing the hard PVC sachets, however this was 42.2% and 80.4% in hard and soft egg baits respectively [29]. Locality also seems to play a role and may be related to access to or competition for food. In the Navajo Nation, 42.2% of dogs offered an egg-bait swallowed the hard capsule, as compared to just 1.5% in Thailand. The dogs in Thailand were fed on a regular basis in contrast to the dogs in the Navajo Nation who devour baits offered without much chewing. In the current study, bait handling times were considerably shorter than both of these studies, which may relate to the use of the soft rather than hard sachet or potentially a consequence of estimating the time the dogs spent chewing as opposed to timing with a stop clock. Unmanaged food waste and feeding by community members are common food sources for free roaming dogs in India [41] and further investigation is required to investigate the impact that this may have on bait consumption and handling time.

Dogs consumed the egg-bait (77.5%) more often than the gravy-bait (68.7%). Interestingly, very similar consumption rates for the egg-bait were reported in the Navajo Nation (77.4%) and Thailand (78.8%) [29,30]. However, only 54.8% of the dogs in India consumed the whole bait compared to 88.3% and 81.2% in the Navajo Nation and Thailand [29,30], which was thought have been due to variation in the ingredients used between the studies and should be possible to overcome with further refinement.

The most important record was whether the data collector observed release of the blue-dyed liquid in the oral cavity, indicating whether the dog would likely have been vaccinated if the sachet had contained ORV. Of dogs that consumed the bait, at least 73.4% were observed to release liquid in the oral cavity in the current study, as compared to 84.5% in Thailand and 89.9% in the Navajo Nation [29,30]. Of all dogs offered an egg-bait where an outcome was recorded, 64.3% were observed with blue-dyed liquid released in the oral cavity in the present study. This clearly underscores the potential of ORV for dogs in India but also emphasizes that ORV is a complementary tool to parenteral vaccination since not all dogs offered a bait will accept it and not all dogs that consume a bait can be considered vaccinated [31]. Evaluation of vaccination coverage subsequently achieved is difficult due to the lower rates of seroconversion as detected by conventional tests, e.g. seroneutralisation tests (Rapid Fluorescent Focus Inhibition Test—RFFIT, Fluorescent Antibody Virus Neutralization—FAVN), used to evaluate parenteral vaccination [42], however, ORV of dogs can increase the vaccination coverage of the dog population above the level needed to achieve the required herd immunity and therefore interrupt the rabies virus transmission cycle among dogs [31,43].

In the current study, there was considerable variation between teams, indicating an inconsistency in the method of bait distribution. The chances of a dog approaching and consuming a bait is highly influenced by the way that the team approach the dog and how they drop/toss the bait. All of the field staff in the study were experienced in CVR methods and data capture using the WVS App however they had comparatively limited experience with the OBH method, which may account for the variation in bait acceptance seen between teams. This finding underscores the need for the development of comprehensive and effective training resources that achieve consistency between teams. The half day training described in this study would need to be followed by a period of field supervision and evaluation to ensure that competence in the distribution method is reached. One of the main advantage of the OBH method as practiced in this study is that even if baits are not accepted, the risk of direct human contact with the vaccine is reduced to negligible levels through recollection of unconsumed baits [44].

The contribution of ORV can be essential for the elimination of dog rabies, especially in areas with a high proportion of free-roaming dogs inaccessible for parenteral vaccination. For example in Bali, Indonesia, free-roaming owned dogs outnumber the restricted owned dogs as 66% to 79% of the owned dogs are free-roaming [10,45]. Furthermore, free-roaming owned dogs in Bali were 2–3 times more likely to be unvaccinated compared to those confined [45] and the majority of dogs were vaccinated by dog catching teams using nets, since most dogs resisted handling even by their owners [10]. Another concern reported from repeated use of net-catching methods was that it can become increasingly difficult to catch free-roaming dogs during subsequent vaccination campaigns [10]. Challenges in accessing dogs for parenteral vaccination were also encountered during mass dog vaccination campaigns in Flores, Indonesia [46]. One of the main reasons identified for owned dogs not being vaccinated was the lack of resources to catch and restrain them by their owners. Intensive vaccination of 70% of the dog population has been demonstrated in focal projects in India, however the challenge of how to efficiently vaccinate large numbers of inaccessible dogs to achieve herd immunity remains. Combining parenteral and ORV methods may help to increase the feasibility of conducting mass dog vaccination at scale in such settings [21]. The egg-bait investigated in this study could provide an effective delivery vehicle for use in these approaches to finally break the deadlock and eliminate dog rabies from large areas.

For a bait to be of potential use for ORV delivery across large areas of India, it must be both possible to produce efficiently in large quantities and it must be widely culturally accepted in both Hindu and Islamic communities. The use of bovine meat products would not be acceptable to the Hindu community, neither would porcine meat products in Islamic communities. Therefore, a non-meat-based bait which can be mass produced locally would be preferable. The egg-bait evaluated in this study could feasibly be produced at mass-scale using local products and is therefore a candidate for further optimisation. Given the slight difference in ingredients used in previous studies, the authors are confident that continued refinement of the egg construct will increase palatability and rate of likely vaccination above those reported in this study.

## 9. Conclusions

This study reports potential bait types for use in delivery of ORV to dogs in India. The use of an effective, well accepted ORV, alongside parenteral vaccination methods may make it possible to more rapidly scale-up mass dog vaccination activities in India, which is urgently needed to reduce the canine rabies burden. The high acceptance rates of the non-meat-based egg-bait indicates the potential for ORV to access a high proportion of free roaming dogs which cannot be readily accessed for parenteral vaccination.

## Figures and Tables

**Table 1:** Table of bait consumption, perforation and oral release of blue-dyed liquid. Percentages in brackets are the percentage of total baits for that bait type.

	Not Consumed		Bait Consumed				
Bait Type	Not Interested	Interested, Not Consumed	Not Perforated	Perforation Unconfirmed *	Perforation Seen, Oral Contact Unconfirmed *	Perforation Seen, Oral Contact Confirmed	Total
**Egg**	37 (17.7%)	10 (4.8%)	13 (6.2%)	16 (7.7%)	14 (6.7%)	119 (56.9%)	209
**Gravy**	36 (18.5%)	25 (12.8%)	34 (17.4%)	11 (5.6%)	13 (6.7%)	76 (39.0%)	195

* Fields where oral release of liquid was not observed, but may have occurred.

## Supplementary Materials

The following are available online at https://www.mdpi.com/2414-6366/4/3/118/s1.
