# Supplementary material for: Development of a Non-Meat-Based, Mass Producible and Effective Bait for Oral Vaccination of Dogs against Rabies in Goa State, India
Source: Trop Med Infect Dis. 2019 Sep 4;4(3):118. doi: 10.3390/tropicalmed4030118 (PMC6789727; doi:10.3390/tropicalmed4030118)
Supplement: Supplementary file 1 [file tropicalmed-04-00118-s001.pdf]

Supplementary materials

Contents

1 Bait acceptance **2**

2 Bait consumption **3**

3 Capsule perforation **4**

4 Bait outcome **5**

5 Vaccination Assessment **6**

6 Dog demographics **7**

7 Model outputs **8**

7.1 Confirmed ‘vaccination’ outcome 8

7.2 Possible ‘vaccination’ outcome 10

8 Bait handling time **12**

8.1 Bait handling time by dogs in groups and alone 13

8.2 Ordinal logistic regression 13

1 Bait acceptance

Table showing bait acceptance for each bait type (k = number of baits by group, n = total number of baits for bait type, lci = lower 95% confidence interval, uci = upper 95% confidence interval).

bait_type	bait_acceptance	k	n	prop	prop_lci	prop_uci
Egg	Ignored	33	209	15.79	11.12	21.45
Egg	Interested	170	209	81.34	75.39	86.38
Egg	Taken by another dog	5	209	2.39	0.78	5.49
Egg	Unknown	1	209	0.48	0.01	2.64
Gravy	Ignored	32	195	16.41	11.50	22.37
Gravy	Interested	158	195	81.03	74.81	86.27
Gravy	Taken by another dog	3	195	1.54	0.32	4.43
Gravy	Unknown	2	195	1.03	0.12	3.66

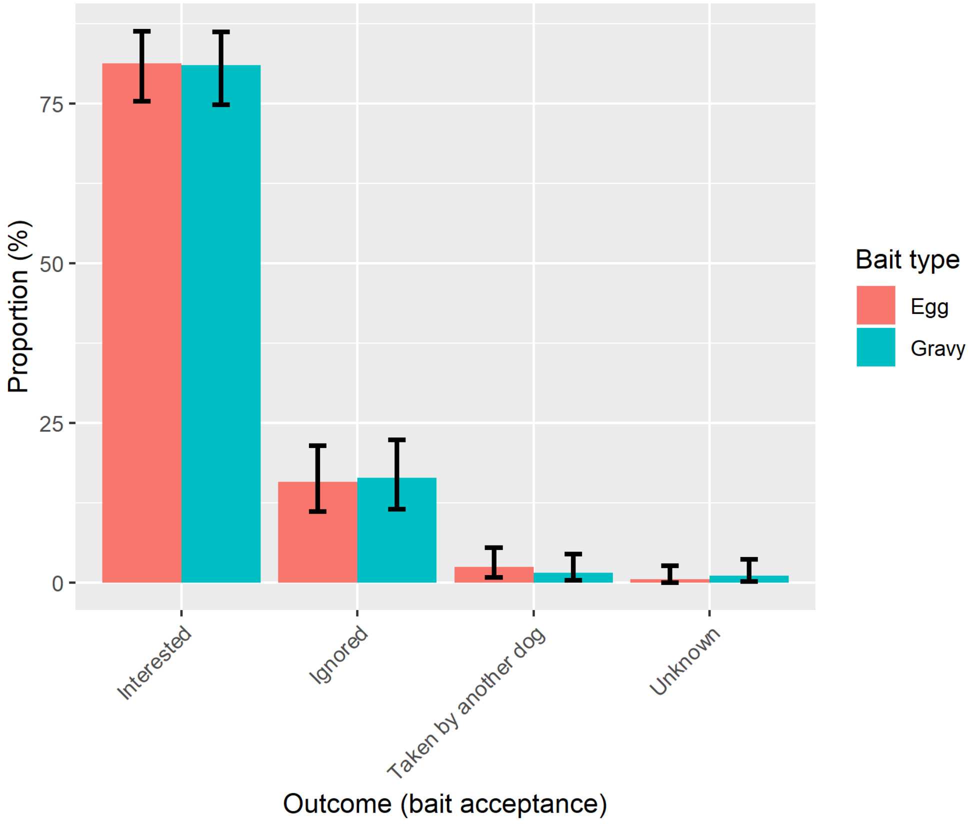

2 Bait consumption

Table showing bait consumption for each bait type (k = number of baits by group, n = total number of baits for bait type, lci = lower 95% confidence interval, uci = upper 95% confidence interval).

bait_type	bait_consumed	k	n	prop	prop_lci	prop_uci
Egg	Not consumed	44	209	21.05	15.73	27.21
Egg	Unknown	3	209	1.44	0.30	4.14
Egg	Yes consumed	162	209	77.51	71.24	82.98
Gravy	Not consumed	59	195	30.26	23.90	37.23
Gravy	Unknown	2	195	1.03	0.12	3.66
Gravy	Yes consumed	134	195	68.72	61.70	75.15

Chart showing the proportion of all dogs offered baits which took the bait into the oral cavity (consumed)

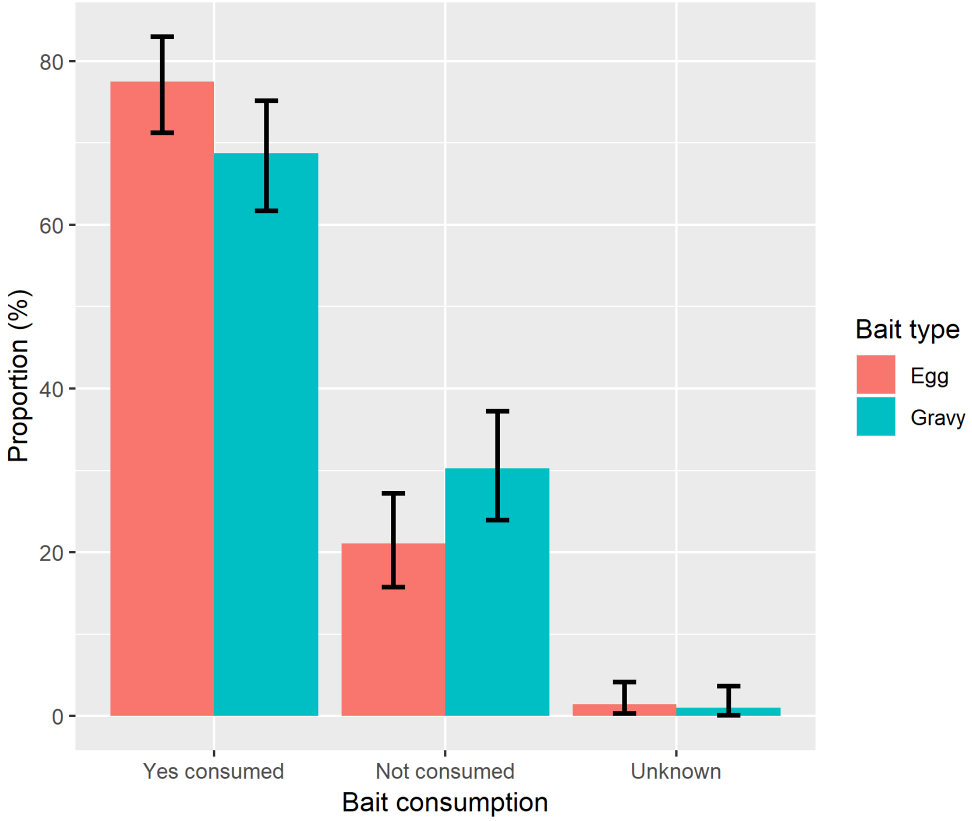

3 Capsule perforation

Table showing capsule perforation outcome for each bait type (k = number of baits by group, n = total number of baits for bait type, lci = lower 95% confidence interval, uci = upper 95% confidence interval).

bait_type	perforate_status	k	n	prop	prop_lci	prop_uci
Egg	Not perforated	13	162	8.02	4.34	13.33
Egg	Unknown	16	162	9.88	5.75	15.54
Egg	Yes perforated	133	162	82.10	75.31	87.67
Gravy	Not perforated	34	134	25.37	18.26	33.61
Gravy	Unknown	11	134	8.21	4.17	14.21
Gravy	Yes perforated	89	134	66.42	57.75	74.34

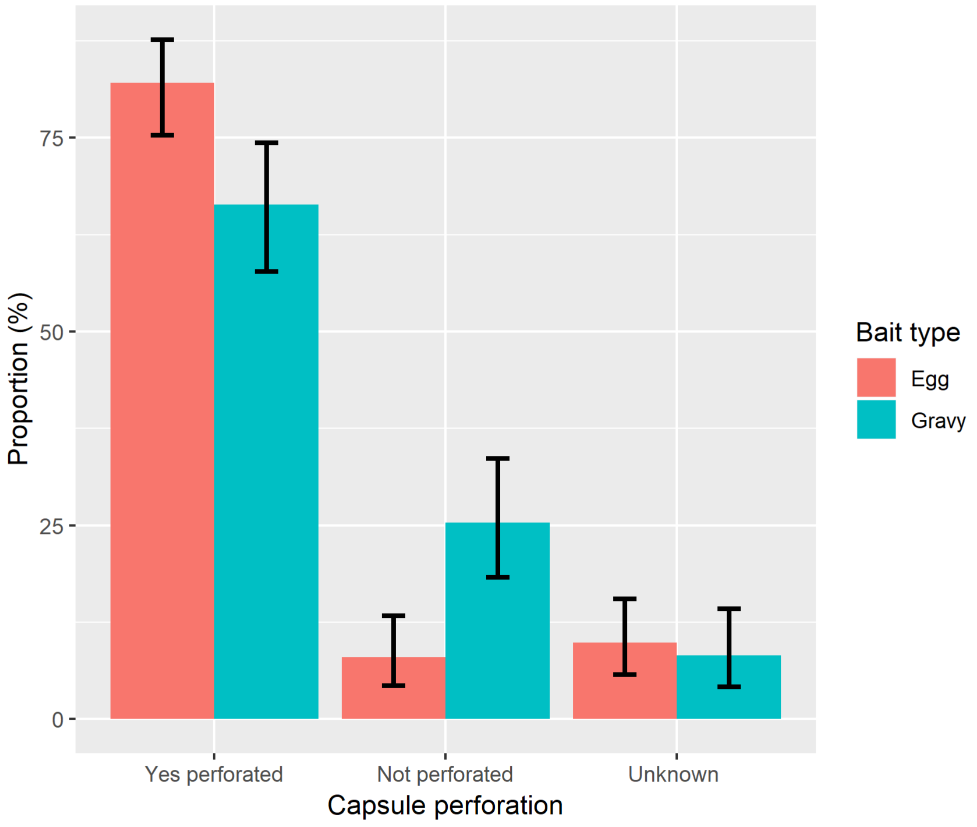

4 Bait outcome

Table showing capsule perforation outcome for each bait type (k = number of baits by group, n = total number of baits for bait type, lci = lower 95% confidence interval, uci = upper 95% confidence interval).

bait_type	capsule_status	k	n	prop	prop_lci	prop_uci
Egg	Discarded	57	162	35.19	27.86	43.07
Egg	Swallowed	97	162	59.88	51.90	67.49
Egg	Unknown	8	162	4.94	2.16	9.50
Gravy	Discarded	85	134	63.43	54.68	71.58
Gravy	Swallowed	47	134	35.07	27.04	43.79
Gravy	Unknown	2	134	1.49	0.18	5.29

Chart showing the perforation status for all dogs consuming baits

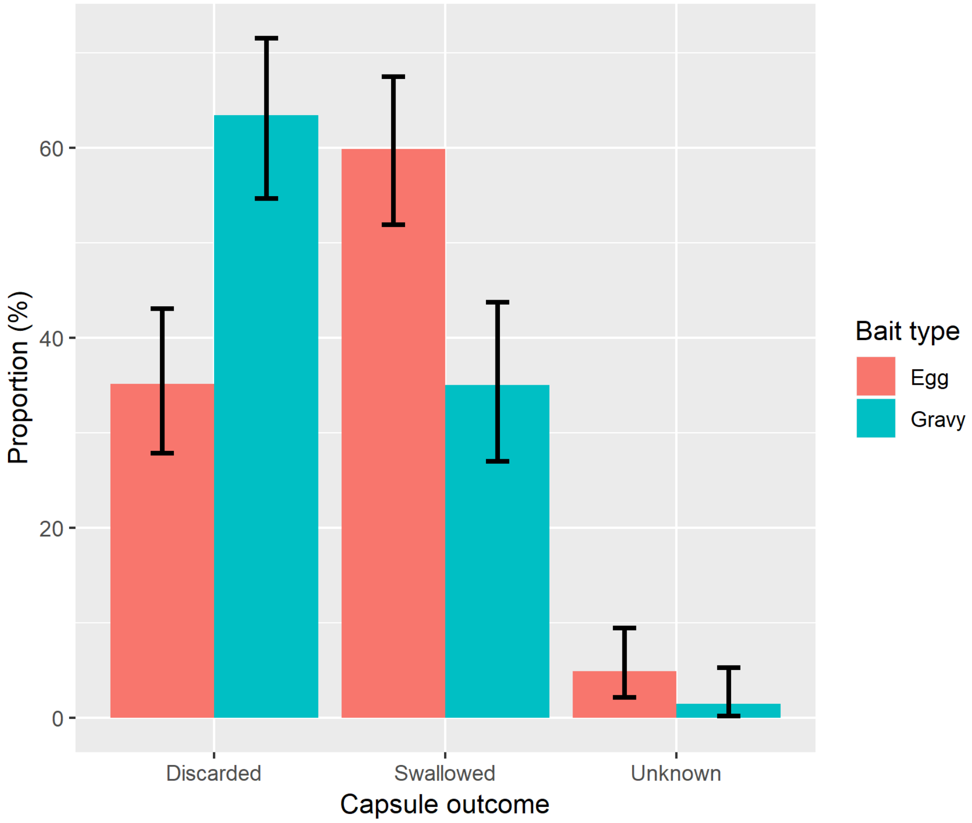

5 Vaccination Assessment

Table showing vaccination assessment outcome for each bait type (k = number of baits by group, n = total number of baits for bait type, lci = lower 95% confidence interval, uci = upper 95% confidence interval).

bait_type	assessment	k	n	prop	prop_lci	prop_uci
Egg	No oral release	64	209	30.62	24.45	37.35
Egg	Unknown	26	209	12.44	8.29	17.69
Egg	Yes oral release	119	209	56.94	49.93	63.75
Gravy	No oral release	97	195	49.74	42.52	56.97
Gravy	Unknown	22	195	11.28	7.21	16.58
Gravy	Yes oral release	76	195	38.97	32.09	46.20

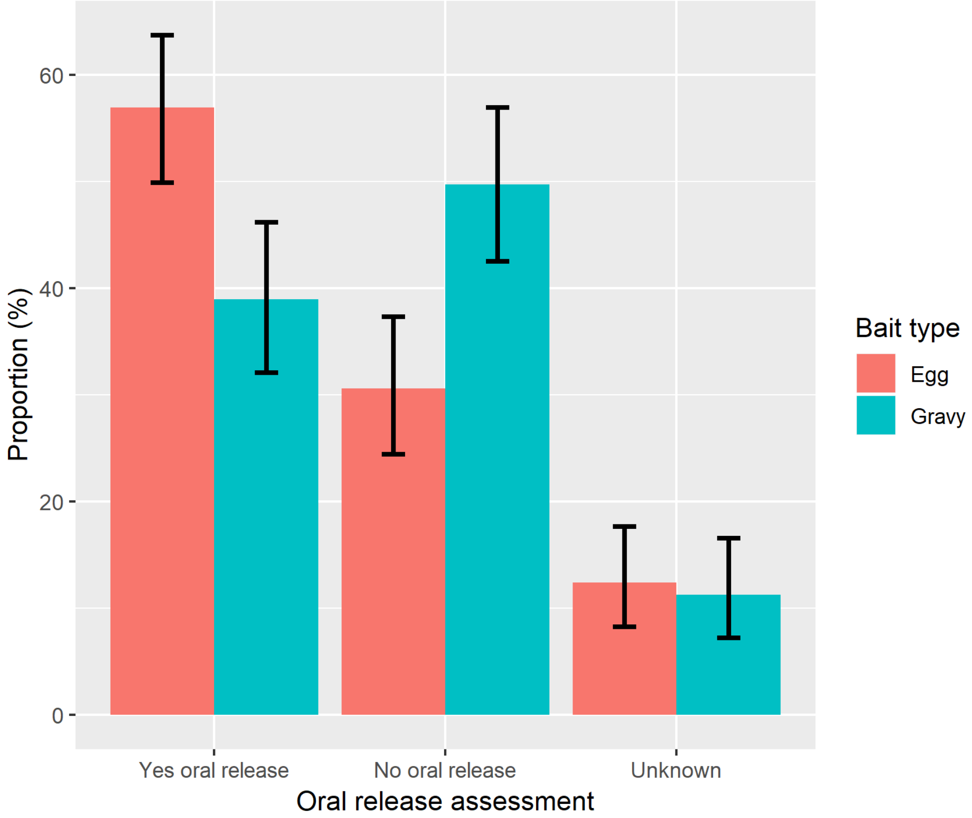

6 Dog demographics

Table showing demographic data for each bait type.

Variable	Egg	Gravy
**Sex**		
female	29	41
male	131	102
unknown	49	52
**Age**		
adult	182	170
juvenile	11	16
puppy	3	1
unknown	13	8
**Size**		
medium	167	156
large	5	5
small	17	19
unknown	20	15
**Ownership**		
owned	NA	1
stray	199	186
unknown	10	8
**Supervision**		
confined	4	1
roaming	200	190
unknown	5	4
**Group**		
multiple	73	62
single	119	118
unknown	17	15

7 Model outputs

7.1 Confirmed 'vaccination' outcome

Multivariable regression model which explored the factors that predicted observation of release of blue-dye liquid in the oral cavity.

variable	estimate	lci	uci	p.value
Bait type: Egg	2.25	1.47	3.43	<0.001
Sex: Female	1.20	0.68	2.11	0.526
Sex: Unknown	0.59	0.33	1.04	0.067
Team: Leopards	0.34	0.20	0.59	<0.001
Team: Rai	0.48	0.29	0.81	0.005

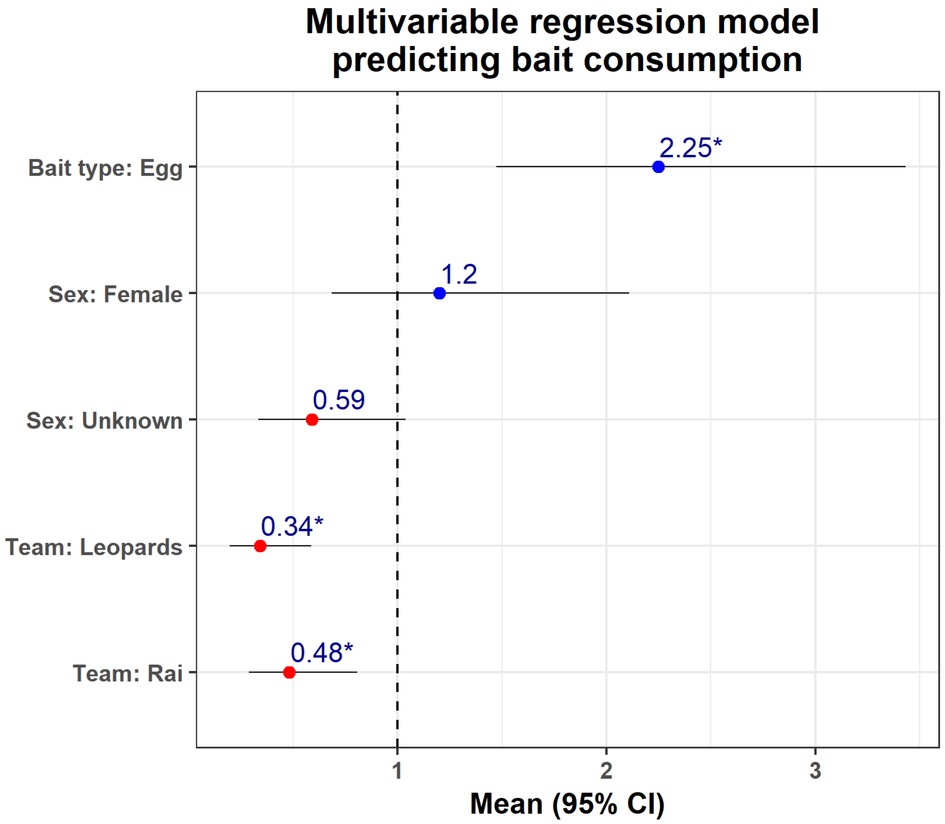

bait_type	prob	lci	uci
gravy	0.37	0.30	0.44
egg	0.57	0.49	0.64

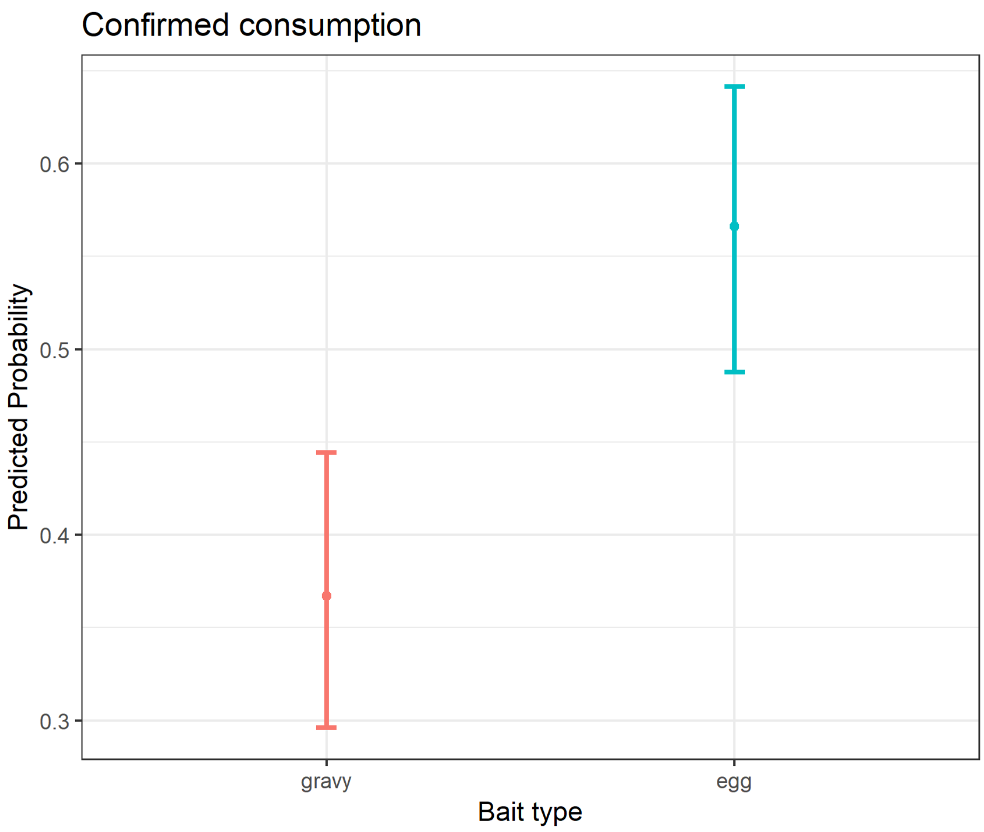
Confirmed consumption
Predicted Probability
0.6
0.5
0.4
0.3
gravy
egg
Bait type

7.2 Possible 'vaccination' outcome

In addition to dogs which were observed to release blue dye liquid in the oral cavity, this group included dogs where the bait was seen to be consumed, but release of blue dye could not observed and yet may still have occurred.

variable	estimate	lci	uci	p.value
Bait type: Egg	2.63	1.71	4.05	<0.001
Sex: Female	1.52	0.83	2.78	0.173
Sex: Unknown	0.52	0.30	0.92	0.024
Team: Leopards	0.81	0.46	1.43	0.475
Team: Rai	0.46	0.27	0.79	0.005

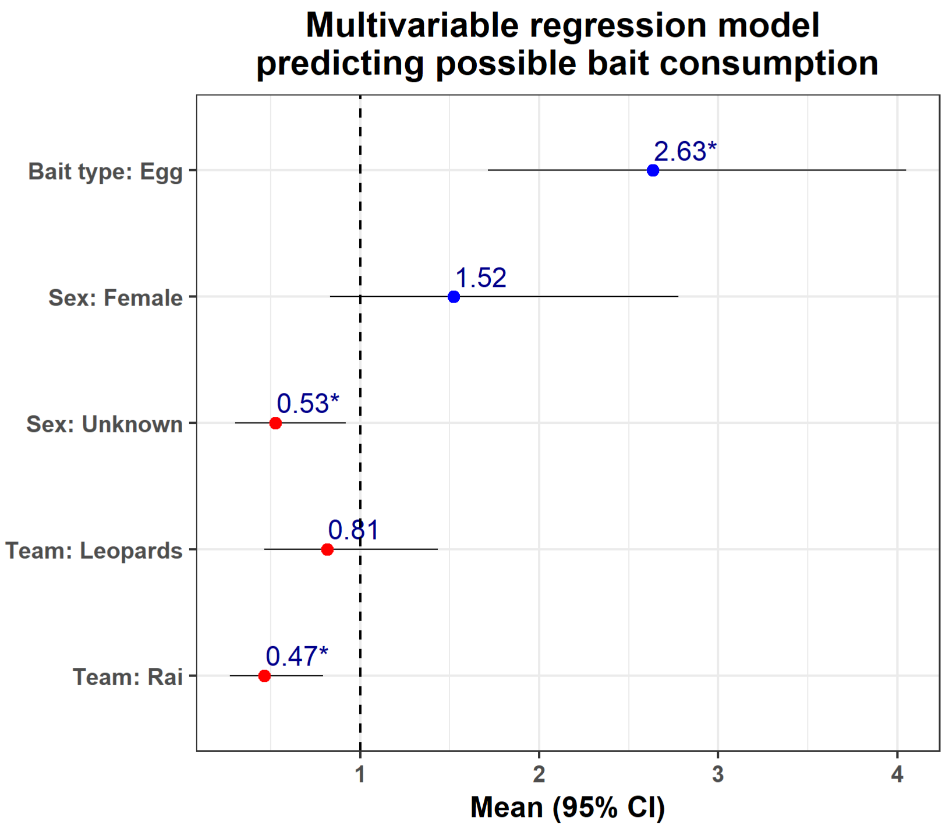

bait_type	prob	lci	uci
gravy	0.50	0.42	0.57
egg	0.72	0.65	0.79

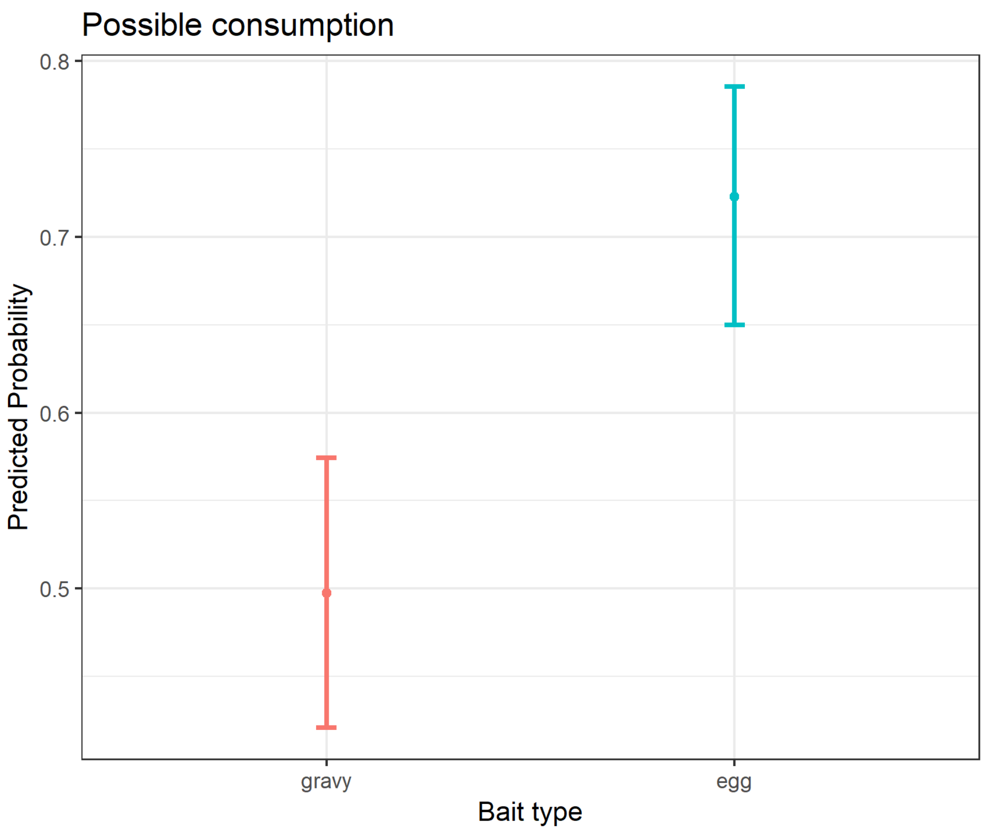
Possible consumption
0.8
0.7
0.6
0.5
Predicted Probability
gravy
egg
Bait type

8 Bait handling time

Table showing bait handling time by bait type

bait_type	chewing_time	k	n	est	lci	uci
gravy	<10 sec	57	128	0.45	0.36	0.54
gravy	10-30 sec	57	128	0.45	0.36	0.54
gravy	30-60 sec	13	128	0.10	0.06	0.17
gravy	>60 sec	1	128	0.01	0.00	0.04
egg	<10 sec	61	150	0.41	0.33	0.49
egg	10-30 sec	64	150	0.43	0.35	0.51
egg	30-60 sec	17	150	0.11	0.07	0.18
egg	>60 sec	8	150	0.05	0.02	0.10

Chart of bait handling time and bait type

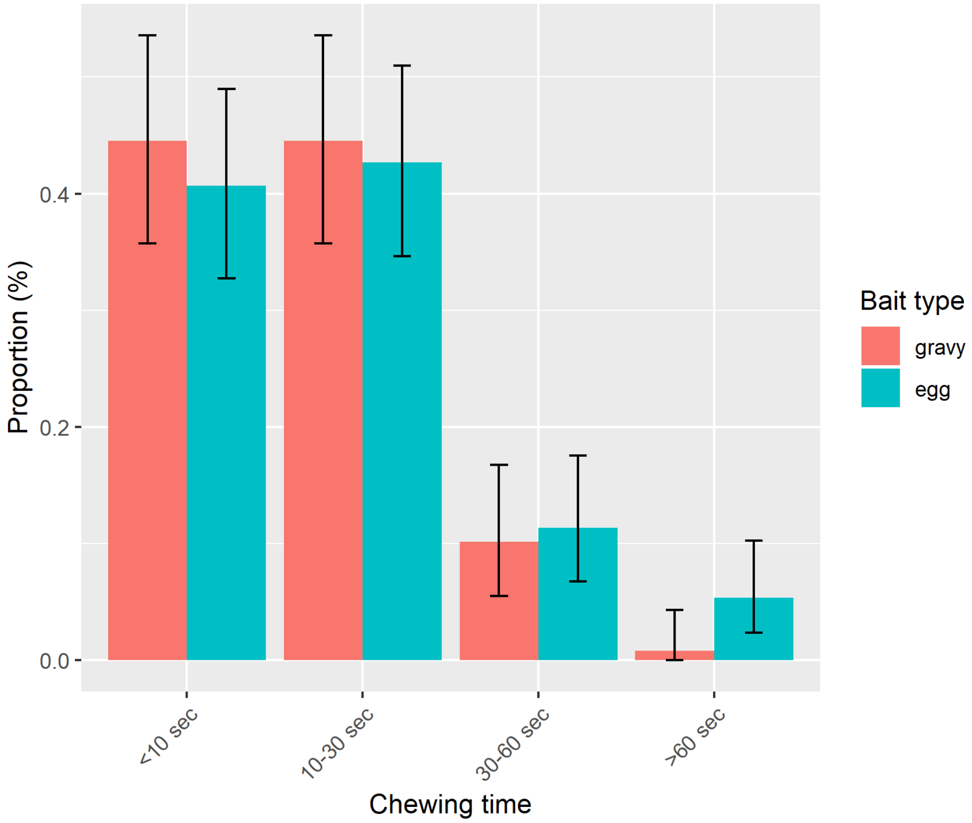

8.1 Bait handling time by dogs in groups and alone

Table summarising chewing time for each bait type for dogs in groups (multiple) or alone (single).

		Egg			Gravy		
Bait consumption	Chewing time	Single	Multiple	Unknown	Single_1	Multiple_1	Unknown_1
Consumed	<10 sec	30	28	3	33	21	3
Consumed	10-30 sec	38	19	7	41	11	5
Consumed	30-60 sec	15	2	0	11	2	0
Consumed	>60 sec	6	2	0	0	1	0
Consumed	unknown	6	4	2	0	4	2
Not consumed		22	18	4	31	23	5
Unknown		2	0	1	2	0	0

8.2 Ordinal logistic regression

Ordinal logistic regression analysis to evaluate the effect of team and the presence of multiple dogs on bait handling time.

bait_type	chewing_time	k	n	est	lci	uci
gravy	<10 sec	57	128	0.45	0.36	0.54
gravy	10-30 sec	57	128	0.45	0.36	0.54
gravy	30-60 sec	13	128	0.10	0.06	0.17
gravy	>60 sec	1	128	0.01	0.00	0.04
egg	<10 sec	61	150	0.41	0.33	0.49
egg	10-30 sec	64	150	0.43	0.35	0.51
egg	30-60 sec	17	150	0.11	0.07	0.18
egg	>60 sec	8	150	0.05	0.02	0.10

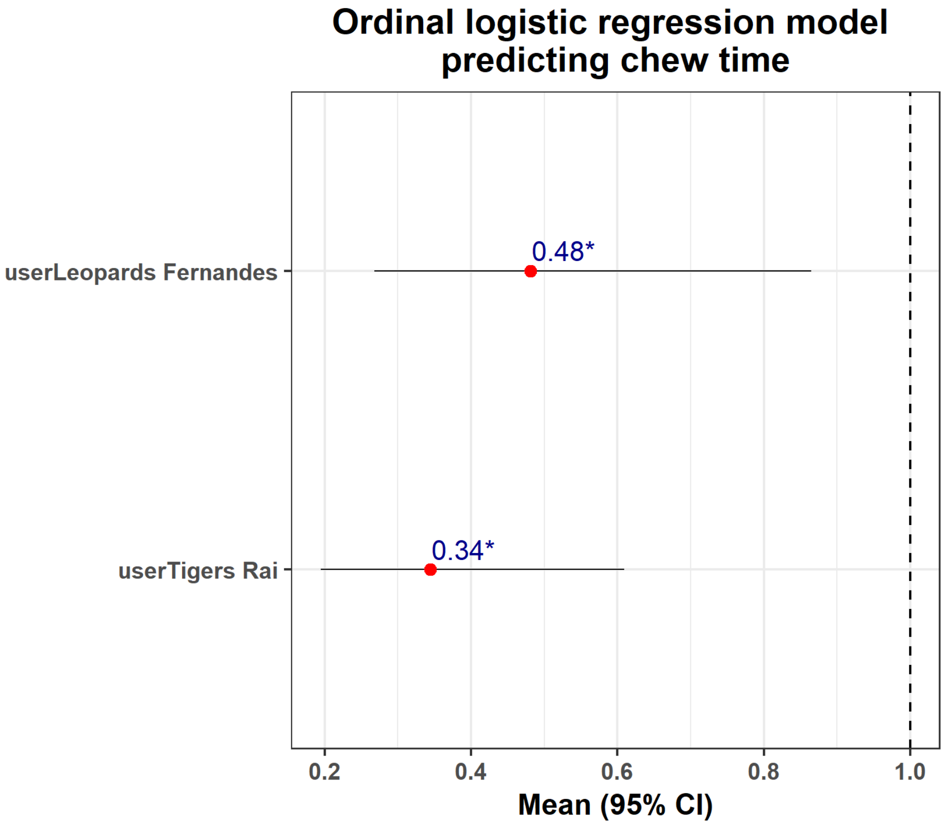
Ordinal logistic regression model
predicting chew time
userLeopards Fernandes
userTigers Rai
0.48*
0.34*
0.2
0.4
0.6
0.8
1.0
Mean (95% CI)
